# Investigating circulating miRNA in transition dairy cows: What miRNAomics tells about metabolic adaptation

**DOI:** 10.3389/fgene.2022.946211

**Published:** 2022-08-23

**Authors:** Arash Veshkini, Harald Michael Hammon, Barbara Lazzari, Laura Vogel, Martina Gnott, Arnulf Tröscher, Vera Vendramin, Hassan Sadri, Helga Sauerwein, Fabrizio Ceciliani

**Affiliations:** ^1^ Institute of Animal Science, Physiology Unit, University of Bonn, Bonn, Germany; ^2^ Research Institute for Farm Animal Biology (FBN), Dummerstorf, Germany; ^3^ Department of Veterinary Medicine, Università degli Studi di Milano, Lodi, Italy; ^4^ Institute of Agricultural Biology and Biotechnology of the CNR, Milan, Italy; ^5^ BASF SE, Lampertheim, Germany; ^6^ IGA Technology Services, Udine, Italy; ^7^ Department of Clinical Science, Faculty of Veterinary Medicine, University of Tabriz, Tabriz, Iran

**Keywords:** miRNA, post-calving, systemic inflammation, negative energy balance, posttranscriptional regulation, immune-related pathways

## Abstract

In the current study, we investigated dairy cows’ circulating microRNA (miRNA) expression signature during several key time points around calving, to get insights into different aspects of metabolic adaptation. In a trial with 32 dairy cows, plasma samples were collected on days −21, 1, 28, and 63 relative to calving. Individually extracted total RNA was subjected to RNA sequencing using NovaSeq 6,000 (Illumina, CA) on the respective platform of IGA Technology Services, Udine, Italy. MiRDeep2 was used to identify known and novel miRNA according to the miRbase collection. Differentially expressed miRNA (DEM) were assessed at a threshold of fold-change > 1.5 and false discovery rate < 0.05 using the edgeR package. The MiRWalk database was used to predict DEM targets and their associated KEGG pathways. Among a total of 1,692 identified miRNA, 445 known miRNA were included for statistical analysis, of which 84, 59, and 61 DEM were found between days −21 to 1, 1 to 28, and 28 to 63, respectively. These miRNA were annotated to KEGG pathways targeting the insulin, MAPK, Ras, Wnt, Hippo, sphingolipid, T cell receptor, and mTOR signaling pathways. MiRNA-mRNA network analysis identified miRNA as master regulators of the biological process including miR-138, miR-149-5p, miR-2466-3p, miR-214, miR-504, and miR-6523a. This study provided new insights into the miRNA signatures of transition to the lactation period. Calving emerged as a critical time point when miRNA were most affected, while the following period appeared to be recovering from massive parturition changes. The primarily affected pathways were key signaling pathways related to establishing metabolic and immune adaptations.

## Introduction

In dairy cows, the transition from pregnancy into lactation often coincides with metabolic stress associated with a negative energy balance (NEB), a result of an imbalance between feed intake and milk production (Drackley, 1999). Several factors such as excessive fat mobilization, impaired liver function, and systemic inflammation during this period may lead to metabolic disorders and also economic losses (Wankhade et al., 2017). Nevertheless, most dairy cows can adapt and maintain their internal organization to deal with transition period challenges, which require an orchestrated array of regulatory mechanisms at the hormonal, metabolic, and immunological levels (Horst et al., 2021).

MicroRNAs (miRNA) are evolutionary conserved small non-coding (approximately 17–24 nucleotides) RNA molecules involved in the post-transcriptional regulation of most protein-coding mRNA, including degradation and translational repression (O'Brien et al., 2018). Since miRNA are involved in almost all metabolic pathways, including energy metabolism, lipid metabolism, insulin sensitivity, and glucose homeostasis (Lin et al., 2020), it is not surprising that their function is crucial during the transition period. As tissue-specific miRNA can enter the circulation system (Barutta et al., 2018) embedded into stable structures such as exosomes and lipoproteins (Condrat et al., 2020), plasma and other body fluids may carry signatures related to the pathophysiological status. Various physiological conditions including heat stress (Lee et al., 2020), different levels of NEB (Hailay et al., 2019), and over-conditioning in dairy cows (Webb et al., 2020), induce changes to the circulating miRNA pattern. In particular, the stage of lactation and transition period affect the miRNA profile. In this regard, the microRNAome of dairy cows’ dry secretions during the first 3 weeks of the dry period revealed differentially expressed miRNA related to reproduction and embryo development (i.e. bta-miR-130b and bta-miR-106a), lactation (i.e. bta-miR-29a, bta-miR-21-3p, bta-miR-130a), as well as inflammation and disease (bta-let-7 family) (Putz et al., 2019).

Markers of the metabolic adaptation during the transition period have been characterized in dairy cows by univariate analyses and, increasingly, via multivariate OMICs technologies (Ceciliani et al., 2018). On the other hand, the post-transcriptional regulation of gene expression has received relatively little attention in this context (Webb et al., 2020). Despite the progress in identifying the origin and the function of miRNA and developing databases, further knowledge of circulating miRNA in dairy cows is still lacking. We, therefore, aimed to characterize the longitudinal changes of the circulating miRNAome profile in dairy cows during the transition period using next-generation sequencing (NGS) and bioinformatics analysis.

## Material and methods

### Experimental design

**FIGURE 1:**
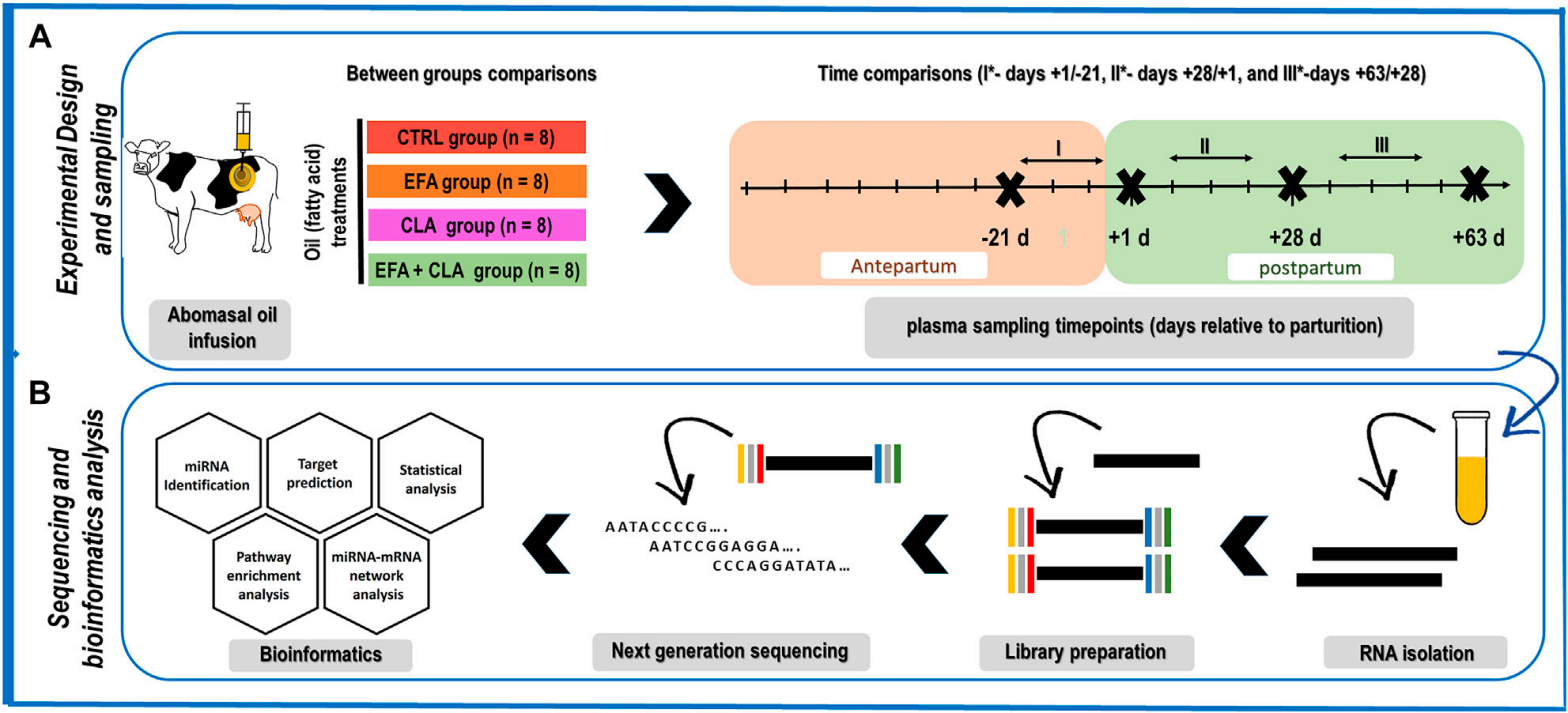
Schematic representation of **(A)** the animal experimental design and sampling time points, consisting of four groups CTRL: control (n = 8), EFA: essential fatty acids (*n* = 8), CLA: conjugated linoleic acid (*n* = 8), and EFA + CLA (*n* = 8), and four plasma sampling (days −21, +1, +28, and +63 relative to parturition). The focus of this study is primarily on time comparisons since the effect of treatments was negligible **(B)** Sequencing analysis and bioinformatics. CTRL: control, EFA: essential fatty acids, CLA: conjugated linoleic acid.

The samples used herein were from an animal experiment described in detail by Vogel (Vogel et al., 2020), performed according to the animal welfare guidelines, and approved by the local authority, i.e. the State Mecklenburg-Western Pomerania, Germany (LALLF M-V/TSD/7221.3‐1‐038/15). In brief, 32 Holstein dairy cows in their second lactation and without clinical signs of disease were housed in free-stall barns with ad libitum access to a corn silage-based total mixed ration (TMR), formulated according to recommendations provided by the Society for Nutrition Physiology (GfE, 2001 (GfE, Gesellschaft für Ernährungsphysiologie (German Society of Nutrition Physiology), 2001), 2008 (GfE, Gesellschaft für Ernährungsphysiologie (German Society of Nutrition Physiology), 2008), 2009 (GfE, Gesellschaft für Ernährungsphysiologie (German Society of Nutrition Physiology), 2009)) and Deutsche Landwirtschaftliche Gesellschaft (DLG, 2013) (DLG (Deutsche Landwirtschafts-Gesellschaft and German Agricultural Society), 2013), from day 63 ante-partum (AP) to day 63 post-partum (PP). The dairy cows were randomly assigned to one of four treatment groups receiving different fat supplements as abomasal infusion: **CTRL** (n = 8; coconut oil, Bio-Kokosöl #665, Kräuterhaus Sanct Bernhard, KG, Bad Ditzenbach, Germany; 38 and 76 g/d in AP and PP), Essential fatty acids (**EFA**, *n* = 8, a combination of linseed oil (DERBY^®^ Leinöl #4026921003087, DERBY Spezialfutter GmbH, Münster, Germany; 39 and 78 g/d in AP and PP), and safflower oil (GEFRO Distelöl, GEFRO Reformversand Frommlet KG, Memmingen, Germany; 2 and 4 g/d in AP and PP), conjugated linoleic acid (**CLA**, *n* = 8, Lutalin^®^; cis-9, trans-11, 10 g/d trans- 10, cis-12 CLA, BASF SE, Lampertheim, Germany; 19 and 38 g/d in AP and PP), and **EFA + CLA**, a combination of linseed oil, safflower oil and Lutalin^®^ from day 63 AP to day 63 PP (Figure 1). The ingredients and the chemical composition of the basal diet were reported elsewhere (Vogel et al., 2020). The present investigation was focused on longitudinal changes only.

### Blood sampling

Four blood samples were collected from each cow on days -21, 1, 28, and 63 relative to calving (Figure 1), immediately after the morning milking and before feeding, using the Vacuette system (Greiner Bio-One International GmbH, Kremsmünster, Austria) containing 1.8 g potassium-EDTA (K3EDTA)/L. Samples were immediately cooled on ice and transferred to the lab within 2 h of collection. The plasma fraction was harvested after centrifugation at 1,500 × *g* (4°C) for 20 min and stored at −80°C until analysis.

### Isolation of total RNA

Total RNA was extracted from 128 plasma samples using the QIAGEN miRNeasy serum/plasma kit (Qiagen GmbH, Hilden, Germany) according to the manufacturer protocol. The miRNeasy Serum/Plasma Spike-In Control (lyophilized *C. elegans* miR-39 miRNA) was used to verify extraction quality and normalize TaqMan qPCR results. The RNA yield and purity were assessed using an Agilent Small RNA kit on a 2100 Bioanalyzer (Agilent Technologies, Santa Clara, CA, United States). In samples with low miRNA concentration (<100 pg/μl), the isolation procedure was repeated using the double initial plasma volume (400 µl).

### Library preparation and next-generation sequencing

The QIAseq miRNA library kit (QIAGEN, Hilden, Germany) was used for library preparation following the manufacturer’s serum/plasma samples instructions. Final libraries were checked with Qubit 2.0 Fluorometer (Invitrogen, Carlsbad, CA) and Caliper (PerkinElmer, Waltham, MA). Afterward, libraries were sequenced on single-end 100 bp mode on the NovaSeq 6,000 (Illumina, San Diego, CA) on IGA Technology Services platform, Udine, Italy (https://igatechnology.com/).

Raw-sequencing data was checked for quality (format conversion to FASTQ, demultiplexing, adapter trimming, and UMI removal) using the Illumina Bcl2fastq2 Conversion Software (version 2.20). A summary of nucleotide sequence data, including FastQC merge reads produced for each sample, sequence quality histograms, per sequence quality scores, per base sequence content, per sequence GC content, per base N content, sequence length distribution, alignment-free estimation of sequences duplication levels, overrepresented sequences, adapter content (multiQC file, version 1.6) is available in the ArrayExpress database (http://www.ebi.ac.uk/arrayexpress) under the “ArrayExpress accession E-MTAB-11725”.

### Sequence analysis

Detected sequences were analyzed with the miRDeep2 (miRDeep2.pl) software package (version 2.0.0.5) (Friedländer, Mackowiak, Li, Chen, Rajewsky) to detect known miRNA and predict putative novel miRNA. MiRDeep2 was fed with the *Bos taurus* miRNA collection available at miRBase (www.mirbase.org (Kozomara et al., 2019)) for known cow miRNA detection. In contrast, the miRBase human, ovine, and goat miRNA datasets were added to support the identification of novel miRNA. Read counts for each known and novel miRNA were compiled using HTSeq-count (Putri et al., 2022).

### Statistical analysis and bioinformatics

The quantified counts were normalized using the TMM method via the edgeR (version 3.12.0) package in R software (Version 4.0). Only miRNA with at least one count per million over at least two samples were considered for the analysis. After normalization, differentially expressed miRNA (DEM) during time were identified by performing generalized linear model (GLM) likelihood ratio tests (glmLRT) using the GLM approach in edgeR to deal with the time series paired effects. Between each two consecutive time points, miRNA with an FDR <0.05 and log2 (FC) > 1.3 were considered as DEM, and visualised by Volcano plot according to their expression (EnhancedVolcano R package). DEM between treatment groups were identified at each time point using the same criterion (FDR <0.05 and log2 (FC) > 1.3). The miRWalk database (2022_01, Bos Taurus, http://mirwalk.umm.uni-heidelberg.de/) was used to compile consensus lists of predicted miRNA targets and to perform gene ontology and KEGG functional enrichment analysis. Network analysis was performed and visualized (yFiles Tree layout) using the combination of miRNet (version 2.0) web-based platform, StringApp, and Centiscape 2.2 in Cytoscape software (version 3.9) (Figure 1). The networks were filtered by two well-established criteria: degree centrality (number of connections with the other nodes) and betweenness centrality (the number of shortest paths connecting the node). The network has been constructed, visualized, and analyzed using the combination of miRanda, miRNet, and Cytoscape. The miRanda database was replaced with the miRWalk database to filter the number of target genes. As yet there is no possibility for target filtration in miRWalk based on Targetscan (http://www.targetscan.org/vert_80/), miRtarbase (https://mirtarbase.cuhk.edu.cn/∼miRTarBase/miRTarBase_2022/php/index.php), and mirdb (http://mirdb.org/faq.html) for dairy cows.

## Results

**FIGURE 2:**
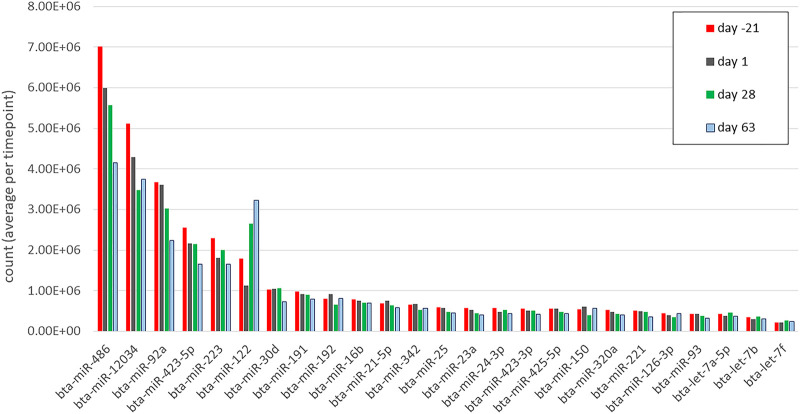
The top 25 miRNAs (by mean counts (million) per time point) in circulation during days −21, 1, 28, and 63 relative to parturition. Different colors correspond to different time points.

In total, 183,772,507 cleaned reads were processed and mapped to the bovine reference genome; in detail, 51,086,403, 46,288,086, 44,508,614, and 41,889,404 reads were processed at days -21, 1, 28, and 63, respectively. From these sequences, 846 miRNA were matched to previously known mature bovine miRNA (equal to 846/1025 = 82.5% of total identified miRNA in cattle until January 2021 (Do et al., 2021), and 846/6808 = 12.42% total theoretical number available in the RumimiR 2022 database, http://rumimir.sigenae.org/). In addition, 1,274 novel miRNA hairpins were identified, including 836 completely novel bovine miRNA (named according to their absolute genomic position), 374 miRNA similar to known *Homo sapiens* miRNA (has-miR), 58 similar to *Capra hircus* known miRNA (ch-miR), and six similar to *Ovis aries* known miRNA (oar-miR) (Supplementary Table S1). The statistical and functional enrichment analyses were limited to 445 cleaned known miRNA with ten counts or more in at least 50% of samples (Supplementary Table S2). Figure 2 represents the 25 miRNA with the highest mean reads (ranging from 2.1 × 10^5^ to 7 × 10^6^) across all samples and time points (sorted by time point). Oil treatments had negligible minor effects on miRNA profile and only one miRNA, bta-miR-1 (log2 (FC) = -5.65, FDR <0.001), was found to be differentially expressed between the CTRL and the CLA group at day -21 AP. There was no other difference in miRNA expression between CTRL, EFA, CLA, and EFA + CLA at days -21, 1, 28, and 63, respectively (Supplementary Table S3). Therefore, data from all four treatment groups were merged to study time-affected miRNA (regardless of the treatment effect).

### Differentially expressed miRNA over time

**FIGURE 3:**
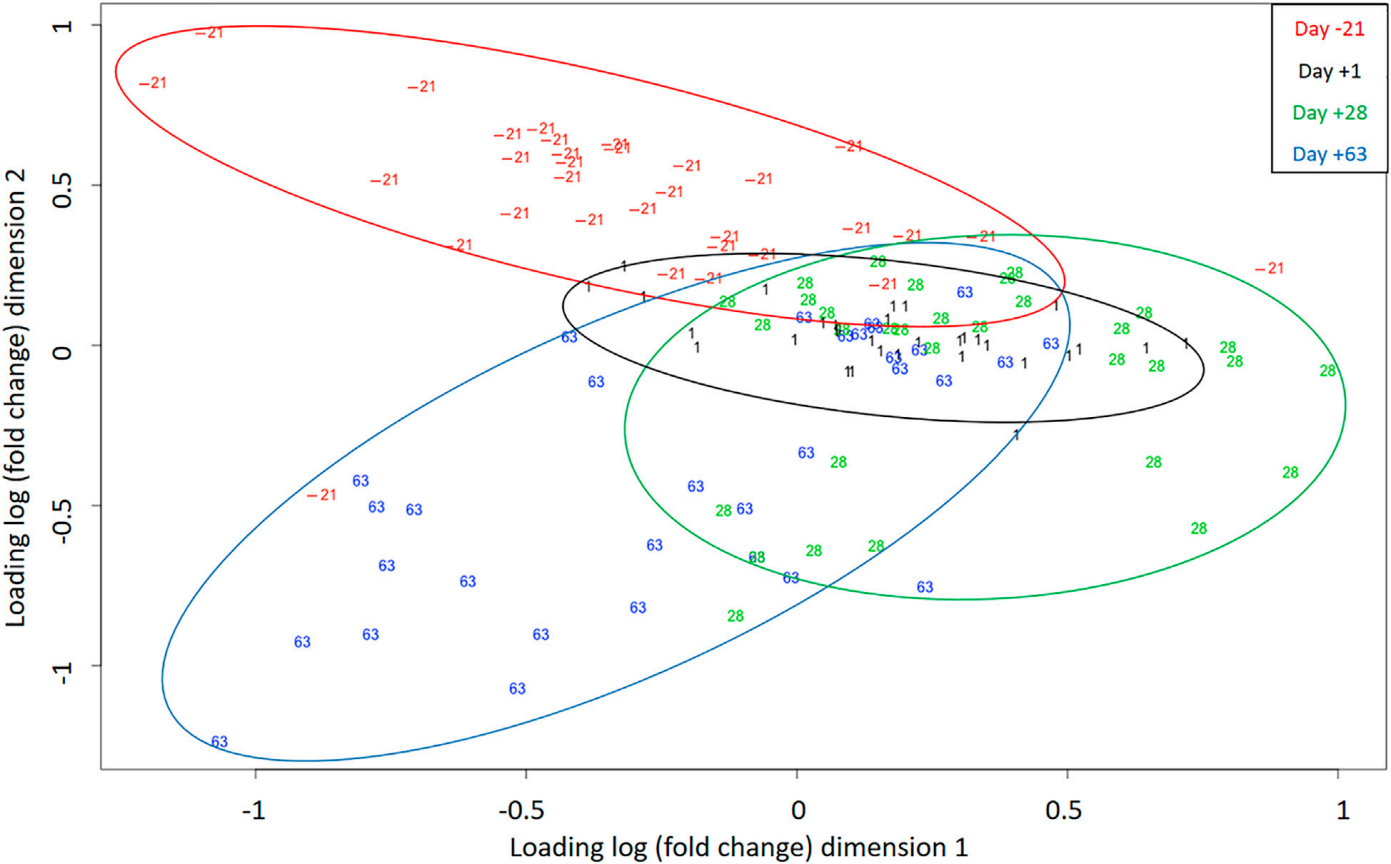
Multidimensional scaling (MDS) plot indicates samples (individual sequencing) separation during days -21, 1, 28, and 63 relative to parturition. Different colors correspond to different time points.

Figure 3 shows the multidimensional scaling (MDS) plot of the individual miRNA profiles obtained on the different sampling days. Using a two-dimensional scatterplot, the distances between samples were approximated by their expression differences. The analysis differentiates the AP (day -21) and PP (days 1, 28, and 63) periods as two well-separated clusters, suggesting that most of the variance in the miRNAome data resulted from the transition to lactation. Even if PP time points partially overlap, clustering according to lactation periods is observable.

### Differentially expressed miRNA during the transition from day −21 to +1 relative to parturition

**FIGURE 4:**
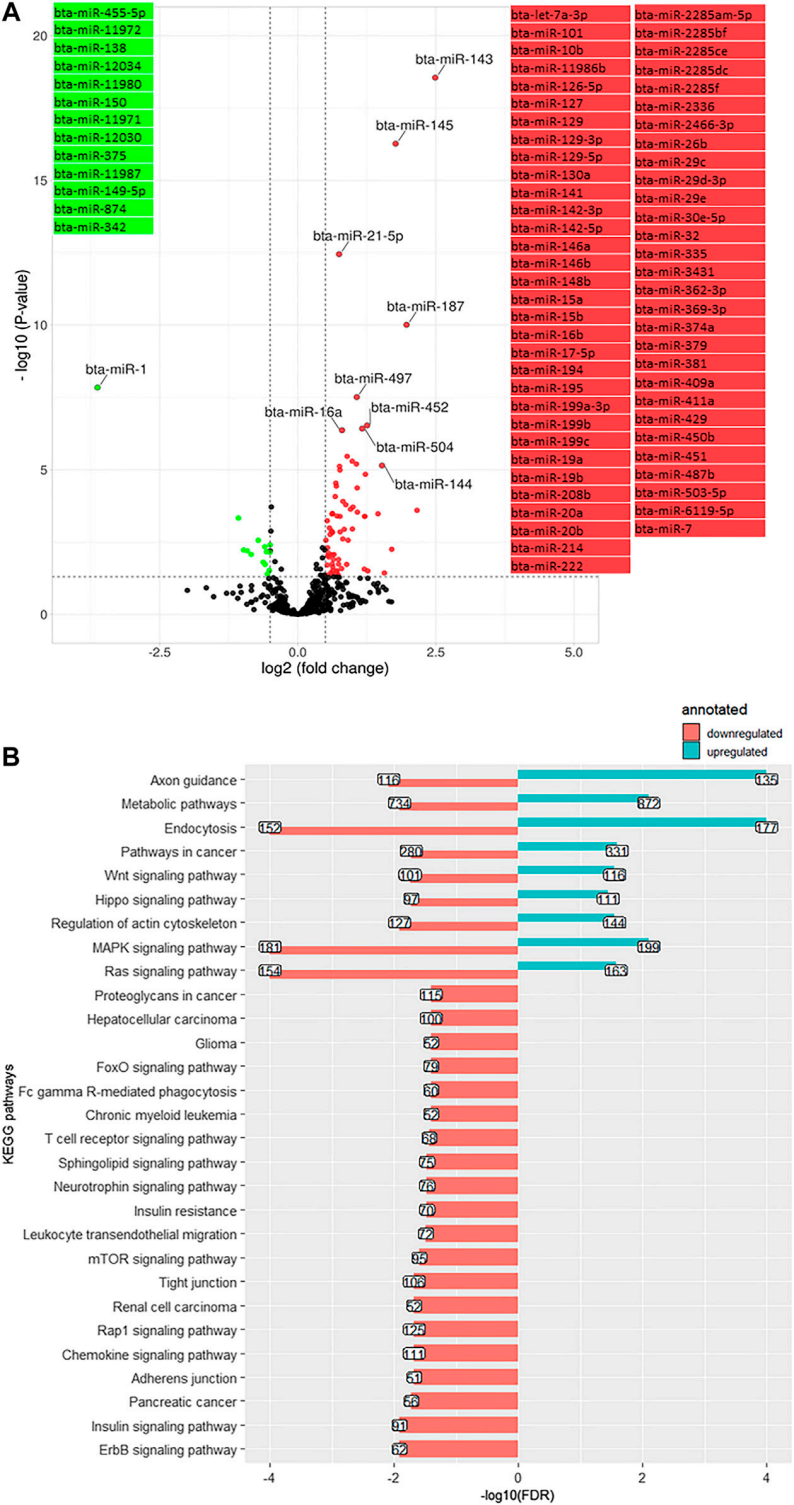
**(A)**. Volcano plot representing differentially expressed miRNA (DEM) between day -21 and +1 relative to parturition (day +1/-21); increased (red dots) and decreased (green dots) miRNA in d+1/d-21 are highlighted (*p* < 0.05 and log2 fold change (FC) > 1.3). **(B)**. KEGG pathways annotated to DEM; Bars indicate proportional to the false discovery rate (FDR) adjusted *p*-value, and the box on each bar represents the gene counts (GC).

Comparing day −21 to 1, 84 miRNA were differentially expressed (log2 (FC) > 1.3, q-value < 0.05), including 14 downregulated and 70 upregulated miRNA (Figure 4A). The most significantly downregulated miRNA was bta-miR-1 (log2 (FC) = −3.62, q-value < 0.001), and the most significantly upregulated was bta-miR-143 (log2 (FC) = 2.5, q-value < 0.001). Using the miRWalk cow database, 25,224 genes were targeted by the downregulated miRNA. These targeted genes were annotated to 29 KEGG pathways (adjusted *p*-value (FDR) < 0.05), mainly related to insulin pathways (insulin signaling pathway bta04910 and insulin resistance bta04931) and cell communication and signaling pathways (mitogen-activated protein kinase (MAPK) signaling pathway bta04010, rat sarcoma (Ras) signaling pathway bta04014, mammalian telomere-binding protein (Rap1) signaling pathway bta04015, endocytosis bta04144, Wnt signaling pathway bta04310, Hippo signaling pathway bta04390, adherens junction bta04520, tight junction bta04530, the mammalian target of rapamycin or mTOR signaling pathway bta04150, sphingolipid signaling pathway bta04071, and T cell receptor (TCR) signaling pathway bta04660) (Figure 4B). The upregulated miRNA predicted targets (50,782 genes) were annotated to nine KEGG (Figure 4B), including the Ras/MAPK pathway, Hippo signaling pathway, and Wnt signaling pathway. Repetition of pathways indicates that a pathway is affected by up and downregulated target genes through possible feedback mechanisms.

### Differentially expressed miRNA between days +1 and +28 of lactation

**FIGURE 5:**
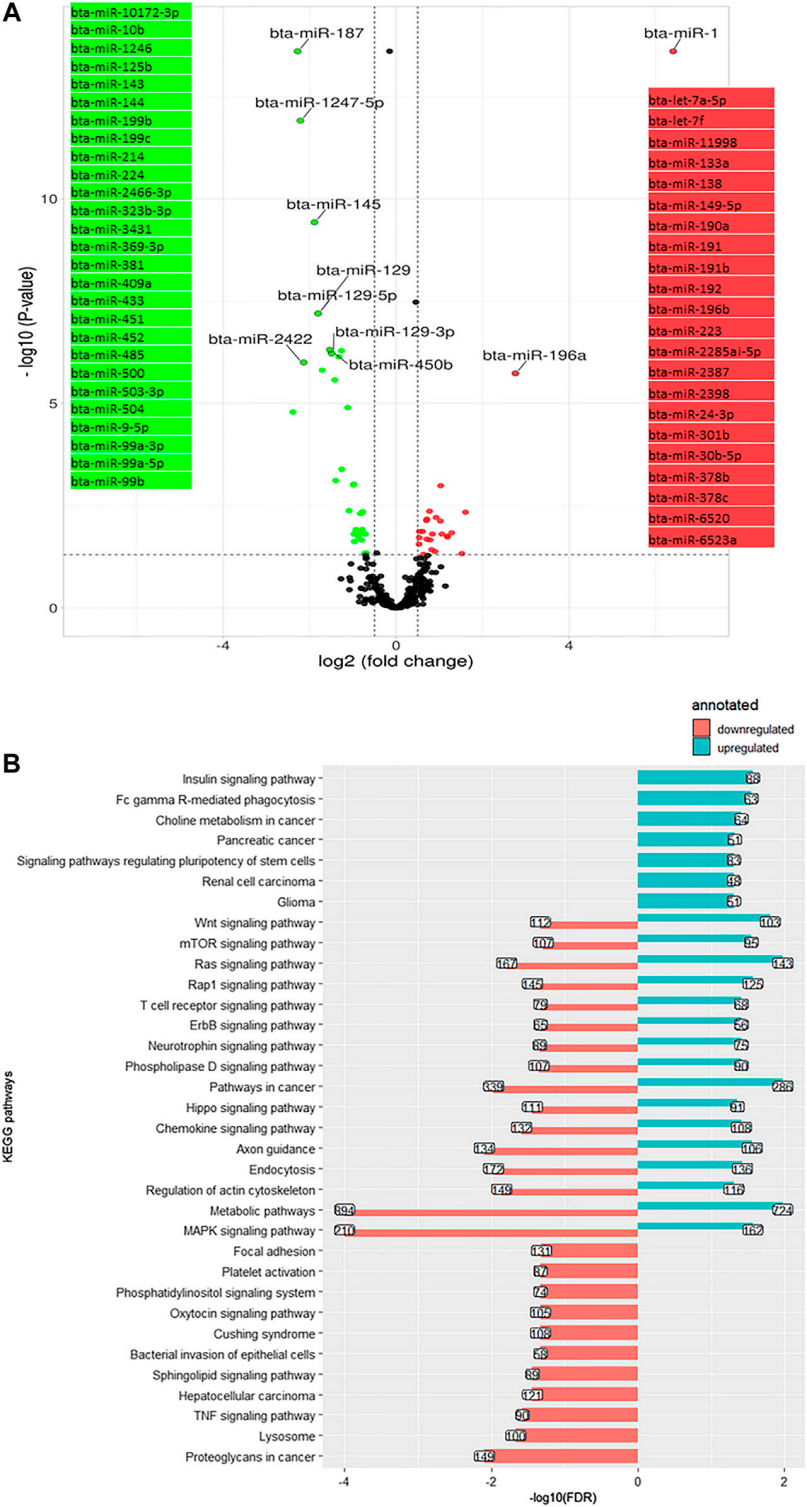
**(A)**. Volcano plot representing differentially expressed miRNA (DEM) between day +1 and +28 relative to parturition; increased (red dots in top right) and decreased (green dots in top left) miRNA in d+28/d+1 are highlighted (*p* < 0.05 and log2 fold change (FC) > 1.3). **(B)**. KEGG pathways annotated to DEM; Bars indicate proportional to the false discovery rate (FDR) adjusted *p*-value, and the box on each bar represents the genes count (GC).

On day 28, compared with day 1, the relative expression was decreased for 35 miRNA and increased for 24 miRNA (Figure 5A). The most significantly downregulated miRNA was bta-miR-187 (log2 (FC) = −2.27, q-value < 0.001), and the most significantly upregulated was bta-miR-1 (log2 (FC) = 6.28, q-value < 0.001). A number of 47,968 genes were predicted as the target of downregulated miRNA annotated to 26 KEGG pathways. The KEGG pathways were mostly signaling pathways including MAPK signaling pathway, Ras signaling pathway, Wnt signaling pathway, Hippo signaling pathway, sphingolipid signaling pathway, TCR signaling pathway, lysosome bta04142, tumor necrosis factor (TNF) signaling pathway bta04668, and oxytocin signaling pathway bta04921, as well as bacterial invasion of epithelial cells bta05100 (Figure 5B). The overexpressed miRNA targeted (prediction) 26,570 genes were related to 23 KEGG pathways, including the Ras signaling pathway, Wnt signaling pathway, MAPK signaling pathway, insulin signaling pathway, mTOR signaling pathway, endocytosis, and TCR signaling pathway (Figure 5B).

### Differentially expressed miRNA on day +28 versus +63 of lactation

**FIGURE 6:**
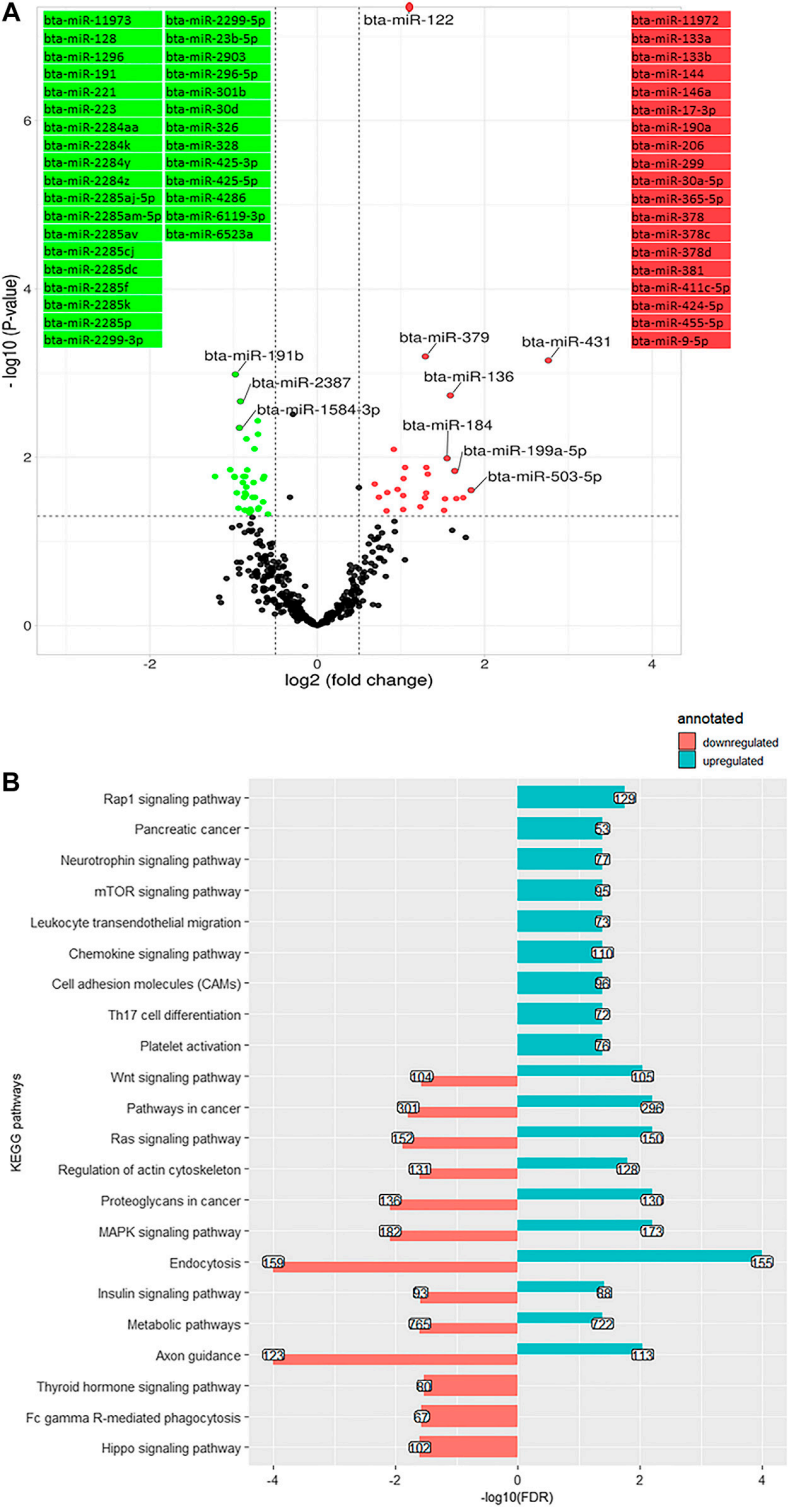
**(A)**. Volcano plot representing differentially expressed miRNA (DEM) between day +28 and +63 relative to parturition; increased (red dots in top right) and decreased (green dots in top left) miRNA in d+63/d+28 are highlighted (*p* < 0.05 and log2 fold change (FC) > 1.3). **(B)**. KEGG pathways annotated to DEM; Bars indicate proportional to the false discovery rate (FDR) adjusted *p*-value, and the box on each bar represents the genes count (GC).

A total of 61 DEM were identified on day 63 compared to day 28, including 35 downregulated and 26 upregulated ones, with bta-miR-122 (log2 (FC) = 0.89, q-value < 0.001) and bta-miR-191b (log2 (FC) = -0.97, q-value < 0.001) being the two extremes (Figure 6A). A number of 33,924 genes were predicted as the target of downregulated DEM which were annotated to 13 KEGG pathways, consisting of a cluster of signaling pathways: MAPK signaling pathway, Ras signaling pathway, Hippo signaling pathway, insulin signaling pathway, Wnt signaling pathway, and thyroid hormone signaling pathway bta04919 (Figure 6B). The overabundant miRNA (putatively targeting 31,531 genes) were annotated to 19 cell communication and signaling KEGG pathways, including MAPK signaling pathway, Ras signaling pathway, Hippo signaling pathway, Wnt signaling pathway, insulin signaling pathway, mTOR signaling pathway, and cell adhesion molecules (CAMs) bta04514 (Figure 6B).

### Pattern identification of commonly affected miRNA during time

**FIGURE 7:**
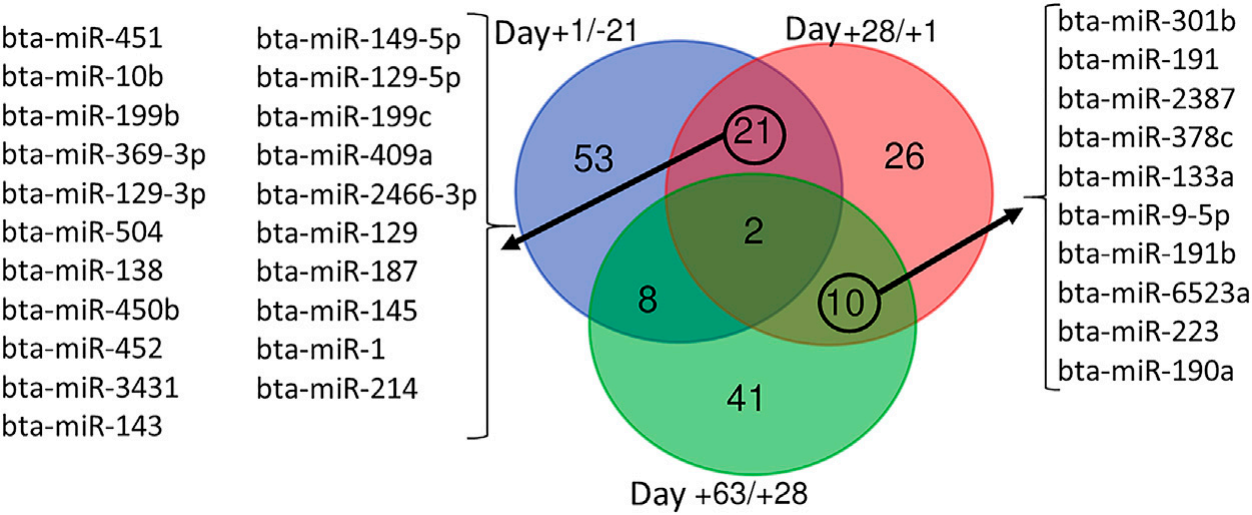
Venn diagram representing the overlap between differentially expressed miRNA during time series comparisons, including Day +1/-21 (blue), Day +28/+1 (red), and Day +63/+28 (green).

**FIGURE 8:**
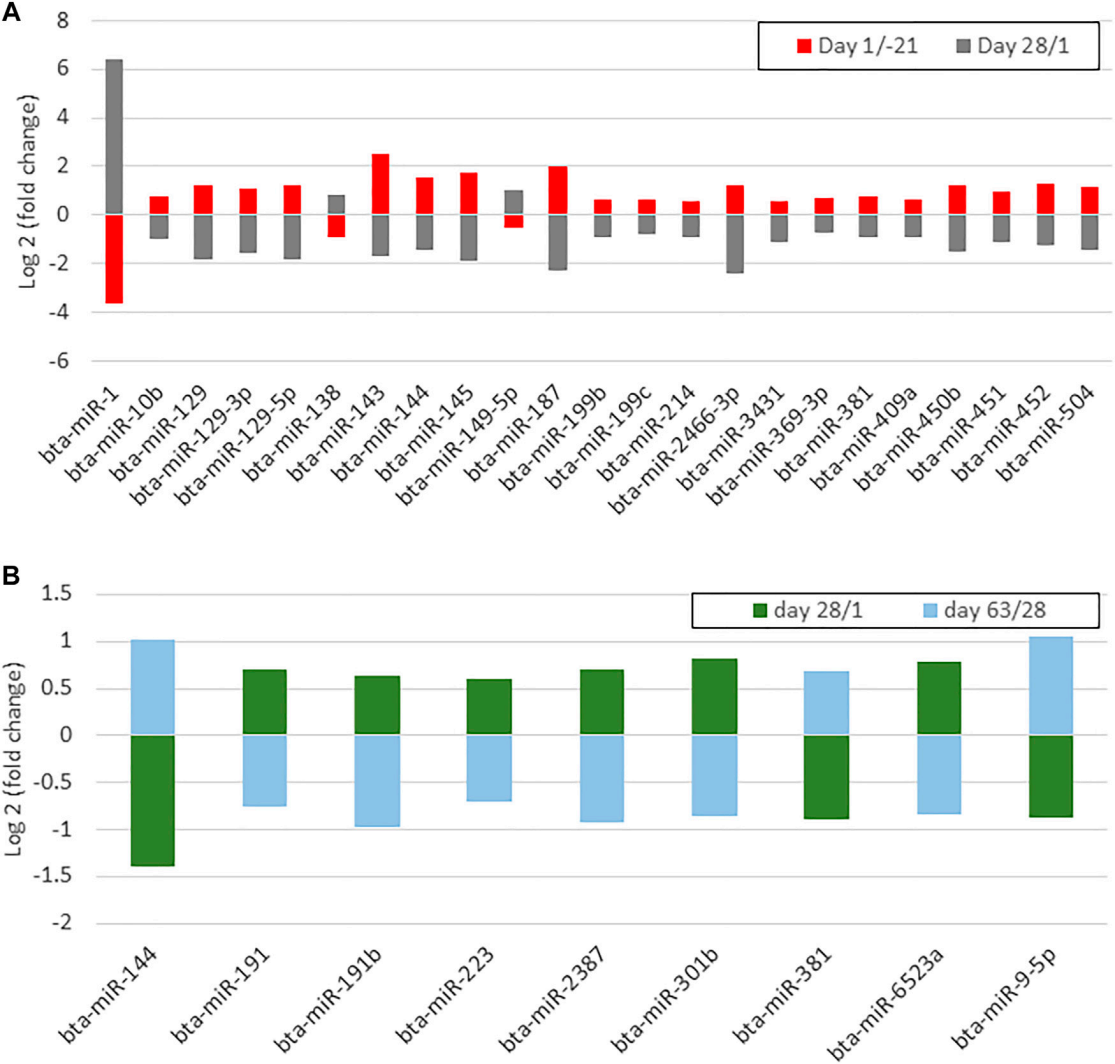
**(A)** A bar chart shows differentially expressed miRNAs whose up- or downregulation occurred on day 1 postpartum and returned antepartum levels on day 28 postpartum **(B)** A bar chart shows differentially expressed miRNAs whose up- or downregulation occurred on day 28 postpartum and returned to day 1 levels on day 63 postpartum.

Venn diagram analysis of DEM across the time points revealed that the expression pattern of specific miRNA was time-dependent (Figure 7). Among time-dependent DEM, a cluster (Figure 8A) was affected at parturition and turned back to initial levels within 4 weeks, while another cluster (Figure 8B) was affected at day 28 PP and returned to parturition level at day 63 PP.

### miRNA-target interaction networks

The biological relevance of miRNA is based on their interaction with their target genes (Lee et al., 2020), although correctly interpreting miRNA-mRNA regulatory pathways is challenging due to numerous potential target genes. Based on the “graph theory” (León and Calligaris, 2017), we analyzed the miRNA-mRNA interaction networks to identify critical nodes and hubs that could act as master regulators, to narrow down the focus of the discussion.

**FIGURE 9:**
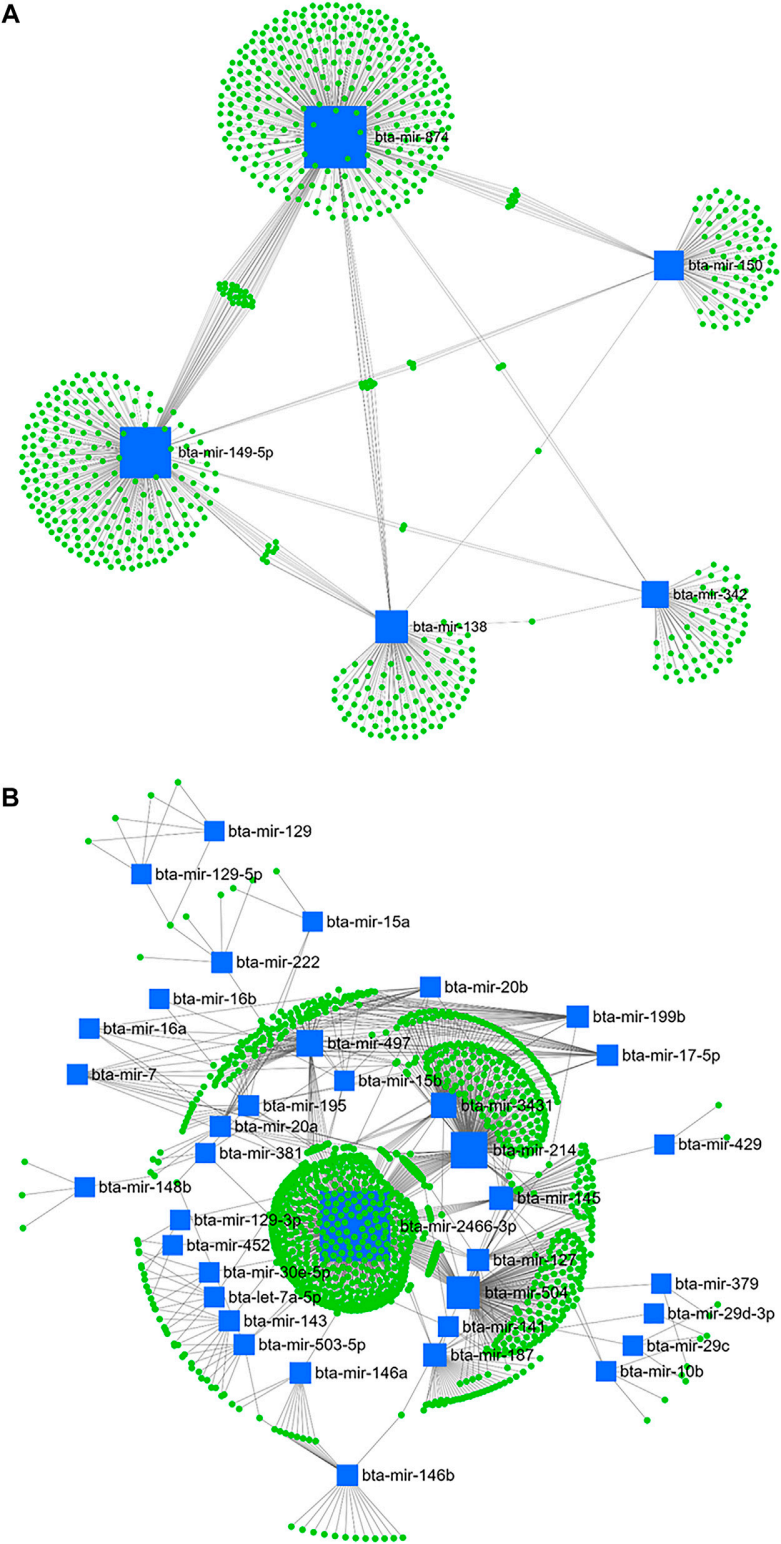
miRNA/mRNA network analysis. The regulation network of downregulated **(A)** and upregulated **(B)** miRNAs and predicted target mRNAs in d+1/-21 is illustrated by Cytoscape software. The nodes represent the mRNA (green), and the boxes represent the miRNA (blue). The box size corresponds to the centrality and betweenness of miRNA in constructing the network.

Accordingly, from day -21 to 1, bta-mir-874, bta-mir-149-5p, bta-mir-138, bta-mir-150, and bta-mir-342 from downregulated (Figure 9A) and bta-mir-2466-3p, bta-mir-214, bta-mir-504, bta-mir-497, bta-mir-3431, bta-mir-145, bta-mir-187, bta-mir-127, bta-mir-199b, bta-mir-146b, bta-mir-143, bta-mir-20a, and bta-mir-195 out of the upregulated miRNA (Figure 9B) were considered as master regulators.

**FIGURE 10:**
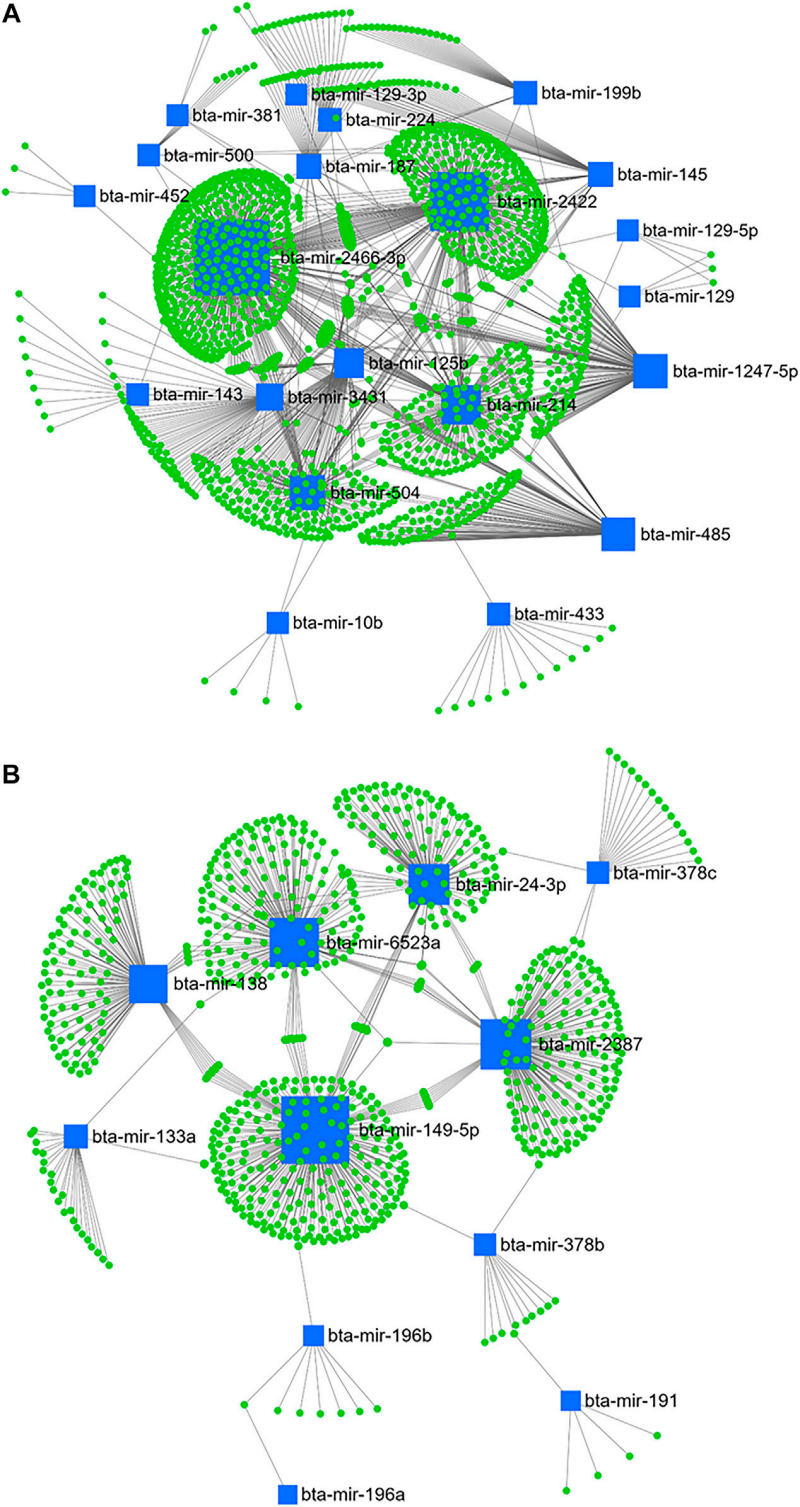
miRNA/mRNA network analysis. The regulation network of downregulated **(A)** and upregulated **(B)** miRNAs and predicted target mRNAs in d+28/+1 is illustrated by Cytoscape software. The nodes represent the mRNA (green), and the boxes represent the miRNA (blue). The box size corresponds to the centrality and betweenness of miRNA in constructing the network.

From day 1–28 PP, bta-mir-2466-3p, bta-mir-2422, bta-mir-214, bta-mir-504, bta-mir-1247-5p, bta-mir-485, bta-mir-125b, bta-mir-3431, bta-mir-145, bta-mir-187, bta-mir-199b, bta-mir-224, bta-mir-433, and bta-mir-143 from the downregulated cluster (Figure 10A), and bta-mir-149-5p, bta-mir-2387, bta-mir-6523a, bta-mir-24-3p, bta-mir-138, bta-mir-133a, bta-mir-378c, and bta-mir-378b from the upregulated cluster (Figure 10B) were selected according to centrality and betweenness.

**FIGURE 11:**
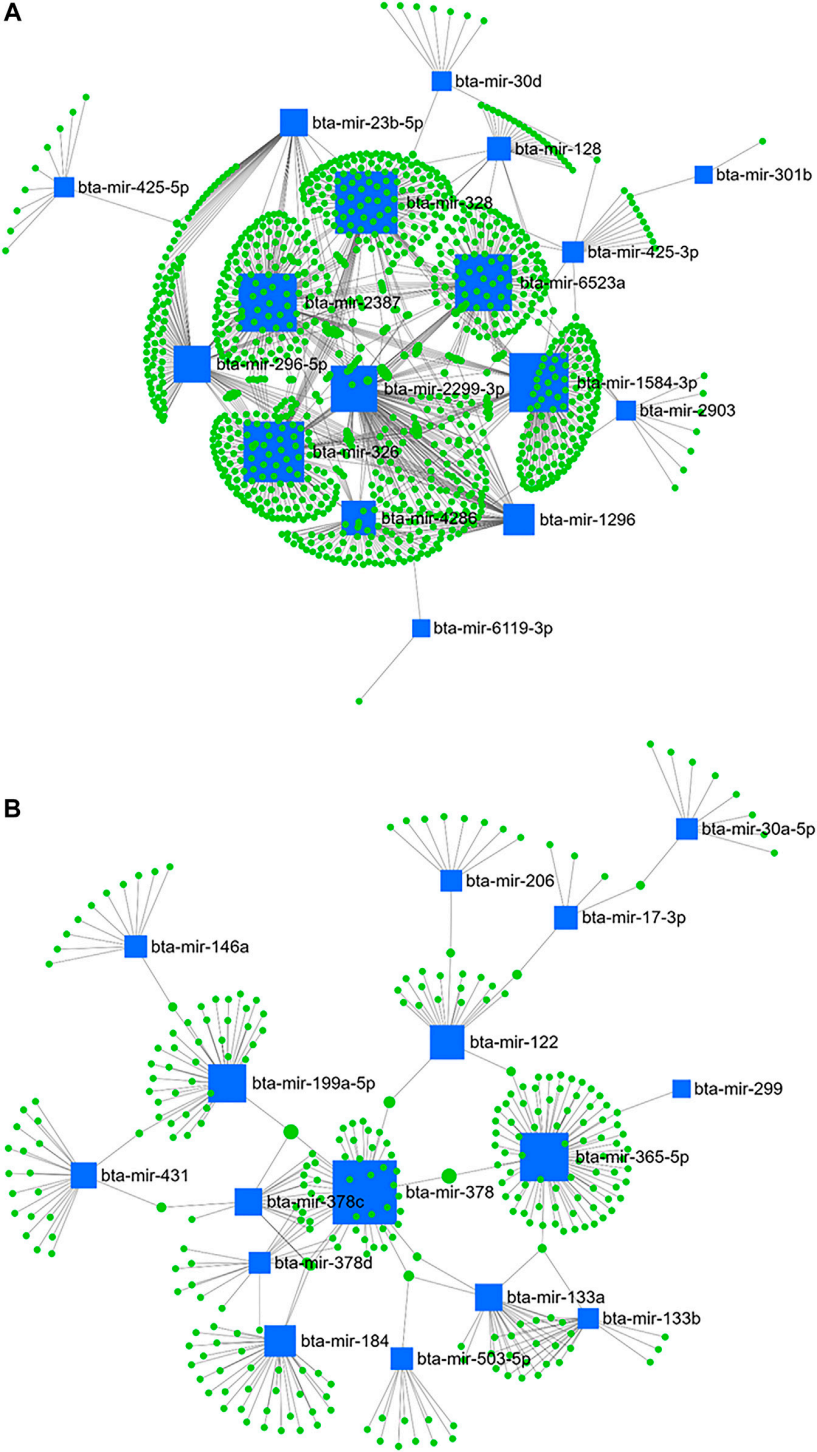
miRNA/mRNA network analysis. The regulation network of downregulated **(A)** and upregulated **(B)** miRNAs and predicted target mRNAs in d+63/+28 is illustrated by Cytoscape software. The nodes represent the mRNA (green), and the boxes represent the miRNA (blue). The box size corresponds to the centrality and betweenness of miRNA in constructing the network.

Furthermore, the highest betweenness was detected between bta-mir-328, bta-mir-326, bta-mir-1584-3p, bta-mir-2387, bta-mir-6523a, bta-mir-2299-3p, bta-mir-296-5p, bta-mir-4286, bta-mir-1296, bta-mir-23b-5p, bta-mir-128, and bta-mir-425-3p from the downregulated miRNA (Figure 11A), and bta-mir-378, bta-mir-365-5p, bta-mir-199a-5p, bta-mir-122, bta-mir-184, bta-mir-378c, bta-mir-133a, bta-mir-431, and bta-mir-378d from the upregulated miRNA (Figure 11B) when comparing day 28 and 63 PP.

## Discussion

MiRNA play an essential role in the post-transcriptional regulation during the endocrine, metabolic, and immune reactions induced by an imbalance between energy requirements and supply during the transition to lactation. Previous studies have shown that severe states of NEB affect the expression of multiple hepatic miRNA and their target genes involved in lipid and glucose metabolism and homeostasis (McCarthy et al., 2010; McCabe et al., 2012; Fatima et al., 2014).

Nevertheless, less information is available regarding the circulating miRNA signature of late gestation and early lactation dairy cows. We used longitudinal miRNAome analysis to characterize DEM at critical time points around parturition and analyze the miRNA-gene network in plasma samples of transition dairy cows to identify candidate miRNA as master regulators of metabolic adaptation. The experimental dairy cows were abomasally supplemented with various fatty acids, and accordingly grouped to CTRL, EFA, CLA, and EFA + CLA. The effects of oil treatment on miRNA expression were negligible, except for the day -21 AP, when bta-miR-1 was differentially expressed between CTRL and CLA groups. No other significant differences were found between the treatment groups. Even though fatty acids treatment altered energy balance in cows of the present study (Vogel et al., 2020), it did not affect miRNA expression or post-transcriptional regulation of energy metabolism. Therefore, the discussion was limited to time-affected miRNA.

### Highly abundant miRNA in plasma of ante and postpartum dairy cows

At day -21 AP and day 1 PP, the top five highly expressed miRNA were bta-miR-486, bta-miR-12034, bta-miR-92a, bta-miR-423-5p, and bta-miR-223, while during days 28 and 63 PP, bta-miR-122 got the position of bta-miR-223 in the top 5. Bta-miR-122 is a hepatic dominant conserved miRNA in dairy cows (Jin et al., 2009) and humans (Jopling, 2012), and is involved in regulating cholesterol and fatty acid metabolism (Wu et al., 2017), but also expressed, albeit at lesser values, in the mammary gland, digestive tract, and extracellular vesicular (serum extracted) exosomes (Sun et al., 2019). When profiling the liver miRNA in early lactation, bta-miR-122 was the highest expressed miRNA in dairy cows with mild and severe NEB (Fatima et al., 2014). Consistent with our results, the dominant expression of bta-miR-486 (highest), bta-miR-92a, and bta-miR-423-5p (among the top five) was previously reported in dairy cows’ blood exosomes (Sun et al., 2019). In mice, there is evidence that miR-486 and miR-92a are involved in hepatic lipid and cholesterol regulatory pathways by targeting sterol-regulatory element-binding transcription factor-1 (SREBF1) and ATP-binding cassette G4 (ABCG4), respectively (Niculescu et al., 2018). Also in humans, these two miRNA are identified in the high-density circulating lipoproteins (HDL) associated with lipid metabolism (Niculescu et al., 2015). Moreover, bta-miR-30d, bta-miR-21-5p, bta-miR-320a, bta-let-7a-5p, bta-let-7b, and bta-let-7f were reported among the most highly expressed miRNA in dairy cows’ dry secretions (Putz et al., 2019). These miRNA have been involved in many aspects of dairy cows’ pregnancy, lactation, inflammation, and disease (Putz et al., 2019).

### Differentially expressed miRNA over time

To ensure comparability, the time points were chosen based on physiological reasons and previously collected data (zootechnical, performance, metabolite, hormone levels, as well as plasma proteomics data) (Vogel et al., 2020; Veshkini et al., 2022), under the identical experimental design. The MDS plot revealed a comprehensive perspective of miRNAome profile in individual samples over time, in which the AP period stands as a separate cluster, while there was a high degree of overlap between days 1 and 28 PP, and day 63 tended to be separated from them and form an independent cluster. Interestingly, the cluster separation was in line with dairy cows’ energy balance (data are shown in (Vogel et al., 2020)), with positive energy balance (day -21) standing out as a distinct cluster, duration of the lowest energy balance (days 1 and 28 PP) as a second cluster, and the last cluster (day 63) appearing to correspond to a period when the energy balance was turning back to positive values. In this regard, miR-143 was differentially expressed in hepatic miRNA profiles of dairy cows at different levels of energy balance and was mainly related to lipid and energy metabolism (Fatima et al., 2014). Therefore, it might be concluded that energy balance is one of the significant determinants of critical pathways in which miRNA are subsequently necessary for initiating and regulating adaptation processes. As recently reviewed (Kinoshita et al., 2016), glucose and lipid metabolism are systemically controlled by a complex orchestrated network containing a plethora of different molecules, such as hormones, lipoproteins, and miRNA. Although there are likely other players involved such as inflammation and immune activation, since in the current design fatty acid treatment significantly affected energy balance (Vogel et al., 2020), but not miRNA expression, which requires further investigation. Therefore, cluster separation was due to time-dependent miRNA expression, in which specific sets of miRNA were probably associated with the physiological state at that time. It should be noted that miRNA expression does not necessarily follow a linear order over time. Thus, there might be other up or downregulated miRNA during the AP and PP periods which were not detected according to our selected time points.

### Differentially expressed miRNA and their associated pathways during the transition period

The transition from gestation to lactation considerably alters miRNA expression, with five times more upregulated miRNA than those downregulated. This might be biologically relevant since most miRNA induce mRNA degradation and translational repression (O'Brien et al., 2018), thereby as part of the adaptation process switching to several organs and tissues’ catabolic status to prioritize milk production over other biological pathways (Veshkini et al., 2022). Although, in the following time points, some of the DEM showed reverse expression, which gradually inverted adaptations to restore normal metabolic and physiological conditions. In this regard, we have previously shown for various metabolites and the proteome that different aspects of the immune system (such as the complement system) (Veshkini et al., 2022) and lipid metabolism including lipoprotein content (Vogel et al., 2020) and apolipoprotein protein abundance (Veshkini et al., 2022) were temporarily downregulated at the time of parturition, and then gradually recovered within the next few weeks after parturition.

The DEM at day 1 PP were annotated to critical immune functions and energy metabolism pathways, including insulin, Ras, MAPK, Wnt, Hippo, sphingolipid, mTOR, and TCR signaling pathways. These signaling pathways are critical to establishing metabolic adaptation at the onset of lactation; therefore, they are strictly controlled. Their dysregulation results in immune and metabolic dysfunction and diseases (Sebastian-Leon et al., 2014). These results align with our recent findings, whereupon metabolic adaptations around calving are most prominent in two distinct but tightly interconnected physiological processes: lipid (energy) metabolism and immune function (Veshkini et al., 2022). The identified adaptation signaling pathways are tightly interconnected and exhibit functions that regulate a diverse array of physiological processes and cellular processes like proliferation, differentiation, transformation, inflammatory responses, apoptosis, and homeostasis in dairy cows (Sigdel et al., 2021). There is also evidence from a human study that circulating and tissue-derived miRNA play a crucial role in the cross-talk between different organs, acting as endocrine and paracrine messengers in intercellular communication (Kiran et al., 2021).

It is important to emphasize that targeted genes may overlap between up and downregulated miRNA; indeed, several pathways were annotated by both up and downregulated miRNA. Information regarding the source or target tissues of DEM is unavailable; therefore, discussing each pathway’s direction according to the direction of miRNA expression seems inefficient. Instead, signaling pathways are discussed according to the literature and our previously published results at the proteome (OMICS based) and metabolite and hormones (classical kit measurements) level from the same experiment, in which the AP period was taken into consideration as the basis and calving as the critical time point for the initiation of the adaptation process.

A major component of immune adaptation was probably triggered by activating the TCR signaling pathway, which activates the NF-κb and downstream signaling cascades that regulate cytokine production, cell survival, proliferation, and differentiation (Hwang et al., 2020). In addition, the TCR signaling pathway initiates intracellular signals essential for T cell development and recruits macrophages for ingesting apoptotic and damaged cells (Moro-García et al., 2018; Shah et al., 2021), which is crucial during the rapid tissue remodeling at calving. Numerous miRNA have been reported to be associated with TCR signaling pathways (Rodríguez-Galán et al., 2018) of which miR-26b, miR-142, miR-146, miR-150, miR-342, and miR-451 were among our DEM.

Specific annotated pathways are downstream targets of the NF-κb pathway, and thus they might have ignited a state of inflammation already underway before parturition. In this regard, intracellular signaling pathways such as Wnt (Jeong et al., 2018), Hippo (Feng et al., 2017), TCR (Adachi and Davis, 2011), Rap1 (Pizon and Baldacci, 2000), and mTOR (Wang et al., 2013) are reportedly related to various physiological and pathological conditions depending on the specific cellular context. The Wnt pathway consists of a large family of highly conserved glycoproteins that cross-talk with MAPK in development, determining cell fate, tissue regeneration, and maintaining tissue homeostasis (Zhang et al., 2014). Previous studies have shown that Wnt and MAPK pathways are essential for normal mammary gland development (Roarty and Serra, 2007; Li et al., 2018) and are involved in immune system-related signaling pathways (Luoreng et al., 2021). These studies reported candidate miRNA including bta-miR-19a, bta-miR-19b, bta-miR-21-5p, bta-miR-29c, bta-miR-143, bta-miR-145, and bta-miR-146b are associated with Wnt signals and MAPK pathway (Li et al., 2018; Luoreng et al., 2021). Mounting evidence from recent human studies also suggested that Wnt is involved in immune signaling, thus regulating immune cell maintenance and renewal, modulation of inflammatory cytokine production, such as NF-κB signaling, and bridging innate and adaptive immunity (Patel et al., 2019; Jridi et al., 2020). Furthermore, Rap1 is shown to function as a transcriptional cofactor that regulates the NF-κB pathway (Yeung et al., 2013).

The current results follow a challenging hypothesis proposed by Bradford et al. (Bradford et al., 2015) as the presence of a sub-acute inflammation during the peripartum period, which has been reviewed later and related to tissue damage and remodeling during this prenatal period in critical organs such as the mammary glands, liver, and adipose tissue (for review, see (Horst et al., 2021)). An earlier study reported that low-grade systemic inflammation, also known as “metabolic inflammation” and “sub-acute inflammation”, plays an important role in developing insulin resistance and hepatic steatosis in mice activating NF-κB signaling pathways (Zeng et al., 2016). As low-grade systemic inflammation in early lactation is a non-infectious inflammatory response, it may activate a type of non-canonical NF-κB that exhibits slower but continuous function (for review, see (Sun, 2011)). Coordinating these signaling pathways develops an immune adaptation process during the transition period in which miRNA play a crucial role (for review, see (Lawless et al., 2014; Chandan et al., 2019)). However, the tissue and cellular level of individual miRNA require further investigation. Further, the current study design did not determine precisely when the systemic inflammation began, reached its peak, and diminished.

Metabolic adaptations are at least partly mediated by alterations in the insulin signaling pathway during early lactation, which is a highly conserved regulatory network coordinating animal metabolism (Zachut et al., 2013). Insulin resistance in the periparturient cow has been ascribed to multiple factors, including low-grade inflammation as one of the primary ones (Rehman and Akash, 2016). Dairy cows in the periparturient period have moderately reduced peripheral tissue insulin sensitivity, which promotes the mobilization of body fat and protein reserves and prioritizes nutrients to the fetus and mammary gland (Zachut et al., 2013). *In vivo* measurements revealed a considerable drop (*p* < 0.001) in plasma insulin levels in the PP period over the AP period (Supplementary Figure S1) and there is a distinct decrease in the insulin release in the PP transition cow (Weber et al., 2016). As a result, we hypothesized that insulin metabolism pathways were partially downregulated. Some of the DEM found herein were previously reported to be associated with the insulin signaling pathway in humans (Nigi et al., 2018). Among those, miR-15b and miR-195 directly regulate the insulin receptor and miR-1 regulates the IGF-1 and IGF1-receptor. Also, miR-122, miR-144, miR-145, miR-146a, miR-214, and miR-375 indirectly affect insulin signaling pathways (Nigi et al., 2018).

The insulin signaling pathways and the sphingolipid signaling pathways, are interrelated: sphingolipid accumulation at the onset of lactation may inhibit insulin signaling and cause insulin resistance in various tissues of dairy cows (McFadden and Rico, 2019), although they are not the sole mediator of insulin antagonism (Petersen and Jurczak, 2016). In that sense, modulating insulin signaling by the sphingolipid signaling pathway and in particular ceramides may considerably impact on regulating glucose utilization, maintaining PP health, and milk production in dairy cows (for review, see (McFadden and Rico, 2019)). Within the sphingolipid pathway, miR-1, miR-101, miR-125b, and miR-128 interact with sphingosine kinases, sphingosine-1 phosphate, and sphingosine-1 phosphate receptors (for review, see (Xu et al., 2021)). The knowledge of sphingolipids in inflammation and endocrine function of dairy cows will continue to grow with the increased accessibility of lipidomics technologies.

In association with the insulin signaling pathway, Ras/Raf/MAPK (rat sarcoma/rapidly accelerated fibrosarcoma/mitogen-activated protein kinases) as major signaling pathways are triggered to transduce signals from the extracellular milieu to the cell nucleus where specific genes are activated to promote gluconeogenesis, lipolysis, re-adjust insulin sensitivity, and regulate the cellular response to various stimuli (Saltiel and Kahn, 2001; Wang et al., 2013). The MAPK family members were also shown to be involved in hepatic and systemic inflammation and apoptosis in dairy cows (Li et al., 2020; Horst et al., 2021), and thus in the initiation of the innate immune system and cytokine receptor-mediated responses, as well as in the adaptive immune system through regulating T and B lymphocyte differentiation (Krzyzowska et al., 2010). Several human miRNA were suggested to be associated with the MAPK signaling pathway (in chronic myeloid leukemia (Chakraborty et al., 2016)), including miR-196a, miR-196b, miR-30a, miR-138, miR-126, miR-221, miR-128, miR-15a, miR-17, and miR-19a that were found as DEM during different time points in the present study.

Also, mTOR is one of the main signaling factors for metabolic adaptation and responds to growth factors, energy and amino acid levels, and cellular stress to regulate transcription, protein and lipid synthesis, sphingolipid biosynthesis, membrane homeostasis, cell cycle progression, endocytosis, and nutrient transport and autophagy (Madrid et al., 2016; Sadri et al., 2016). The mTOR and MAPK signaling pathways may cross-talk to maintain essential cellular functions (Madrid et al., 2016) and probably direct protein synthesis toward the mammary gland and, thus, milk production in early lactating dairy cows. In accordance, a recent study on dairy goats suggested the involvement of the AMPK-mTOR pathway in mammary milk protein synthesis (Cai et al., 2020). The DEM identified herein were predominantly involved in signaling cross-talk regulatory mechanisms that determine energy homeostasis as a part of metabolic adaptations.

As discussed earlier, the expression level of several DEMs was reversed within a few weeks of lactation and then returned to AP expression, which resulted in normalized insulin, mTOR, and Ras signaling pathways. These results are consistent with our previous proteomics findings in which indicators of metabolic and immune adaptation such as acute phase proteins (haptoglobin, lipopolysaccharide-binding protein, alpha-2-Heremans-Schmid glycoprotein, and adiponectin) and various complement proteins at the onset of lactation were restored within four to 9 weeks of lactation (Veshkini et al., 2022).

Beside those affected pathways, the TNF signaling pathway was annotated by downregulated miRNA on day 28 PP. The NF-κB pathway is activated through TNF signalling pathways, producing various proinflammatory cytokines (including TNF-α). Moreover, lipoprotein lipase is inhibited thus decreasing fatty acid uptake and lipogenesis, and increasing lipolysis in adipose tissue (Kim et al., 2014). Conversely, TNF-α promotes the hepatic expression of SREBP1c, a major regulatory transcription factor in hepatic lipogenesis and the development of hepatic steatosis in humans (Kim et al., 2014). In addition, TNF-α, besides being a well-known major proinflammatory cytokine, induces in part insulin resistance through phosphorylation of Jun and insulin receptors (Kim et al., 2014). Accordingly, a possible explanation could be that early lactation inflammation induces TNF-α, affecting downstream pathways, including insulin signaling pathways. Then, TNF signaling was also reduced in conjunction with attenuated systemic inflammation at 28 days PP.

### Determination of key miRNA and their target mRNA in regulatory pathways

As discussed earlier, a group of miRNA had a time-dependent expression including overexpression of bta-miR-10b, bta-miR-129 family, bta-miR-143, bta-miR-144, bta-miR-145, bta-miR-187, bta-miR-199b, bta-miR-214, bta-miR-369-3p, bta-miR-381, bta-miR-409a, bta-miR-450b, bta-miR-451, bta-miR-452, bta-miR-504, and bta-miR-2466-3p and downregulation of miR-1, bta-miR-138, and bta-miR-149-5p at parturition. These miRNA returned to AP expression after 28 days. During the transition from day 1–28 PP, another cluster, including bta-miR-133a, bta-miR-144, and bta-miR-378c, was downregulated, whereas bta-miR-9-5p, bta-miR-190a, bta-miR-191, bta-miR-191b, bta-miR-223, bta-miR-301b, bta-miR-381, bta-miR-2387, and bta-miR-6523a were upregulated. These miRNA regained their parturition levels on day 63 PP. Based on bioinformatics analysis, time-dependent DEM and their predicted target genes have been annotated to insulin secretion, sphingolipids, TNF, NF-κB, B cell receptor, and MAPK signaling pathways, the key indicators in determining metabolic and immune adaptation success.

We determined which miRNA are the central hub of those enriched pathways using miRNA-mRNA network analysis. Considering time-dependent expression and network analysis, bta-miR-138, miR-149-5p, bta-miR-214, bta-miR-504, bta-miR-2387, bta-miR-2466-3p, and bta-miR-6523a were selected as the central hub regulating annotated signaling pathways. Significant positive correlations were observed between bta-miR-504 and bta-miR-2466-3p (r = 0.51, *p*-value < 0.001), and bta-miR-504 and bta-miR-214 (r = 0.6, *p*-value < 0.001) (Supplementary Figure S2). According to bioinformatics analysis, these miRNA and their target genes are among the key regulators of the NF-κB signaling pathway, insulin secretion, sphingolipid, TNF, B cell receptor, and MAPK signaling pathways. Several target genes were common to these signaling pathways including, MAPK3, MAPK14, MAP3K14, MAP2K2, JUN, MYD88, NRAS (neuroblastoma RAS viral (V-ras) oncogene homolog), FOS, IKBKG (inhibitor of nuclear factor-kappa B kinase regulatory subunit gamma), IKBKB (inhibitor of nuclear factor-kappa B kinase subunit beta), and RELA (Supplementary Figure S3), which were the key genes in previously annotated metabolic and immune pathways. Also, protein-protein interaction network analysis revealed that all these genes are biologically connected (Supplementary Figure S4). Overall, bioinformatics provided a subset of miRNA considered the regulatory hub for metabolic and immune adaptation processes, which can be used as candidates to assess the metabolic health of dairy cows during the transition period. Nevertheless, future studies should focus on the specific role of these miRNA in metabolic disorders.

## Conclusion

Investigating circulating miRNA revealed novel insights into miRNA signatures associated with metabolic and immune adaptation in late gestation and early lactation dairy cows. There were time-dependent differences in the expression of specific miRNA, in which the calving effect predominated, such that their expression was differential during the parturition time and returned to normal after a few weeks (days 28 and 63 PP). Calving prevailing was partially caused by DEM associated with a state of systemic inflammation. Bioinformatics analysis suggested that metabolic adaptation initiated by systemic inflammation, and DEM orchestrates critical signaling pathways, including TCR, NF-κB, MAPK, and insulin, which facilitate cross-talk between energy metabolism and immunity in target tissues across the whole body. In particular, the miRNA-mRNA network analysis revealed circulating DEM including bta-miR-138, miR-149-5p, bta-miR-2466-3p, bta-miR-214, bta-miR-504, and bta-miR-6523a and their targeted genes as central hubs that regulate key signal transduction pathways associated with energy homeostasis and immune response in transition dairy cows. However, a more in-depth analysis of the identified miRNA and their target genes is required to develop them as biomarkers of metabolic homeostasis.

## Data Availability

The data presented in the study are deposited in the ArrayExpress database (http://www.ebi.ac.uk/arrayexpress) repository, accession number E-MTAB-11725.
