# Supplementary figures and images for: Investigating circulating miRNA in transition dairy cows: What miRNAomics tells about metabolic adaptation
Source: Front Genet. 2022 Aug 23;13:946211. doi: 10.3389/fgene.2022.946211 (PMC9445238; doi:10.3389/fgene.2022.946211)

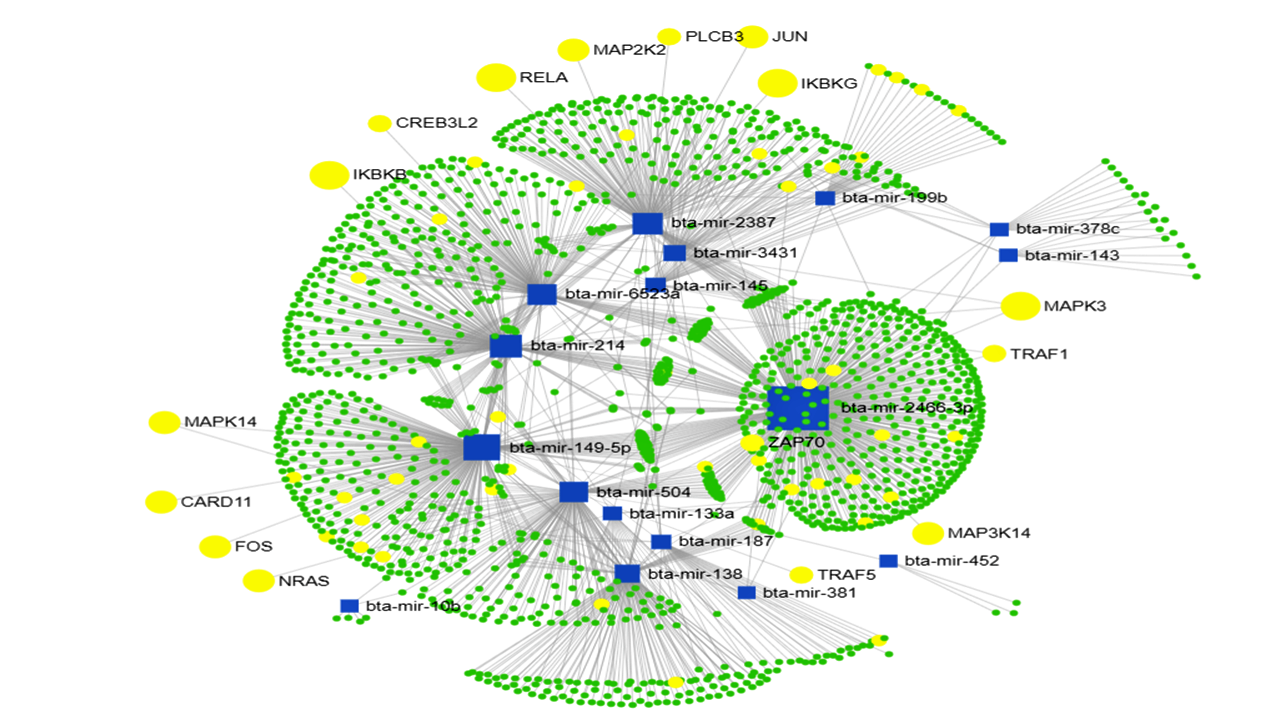

Supplement: Supplementary file 2 [file Image3.TIF]

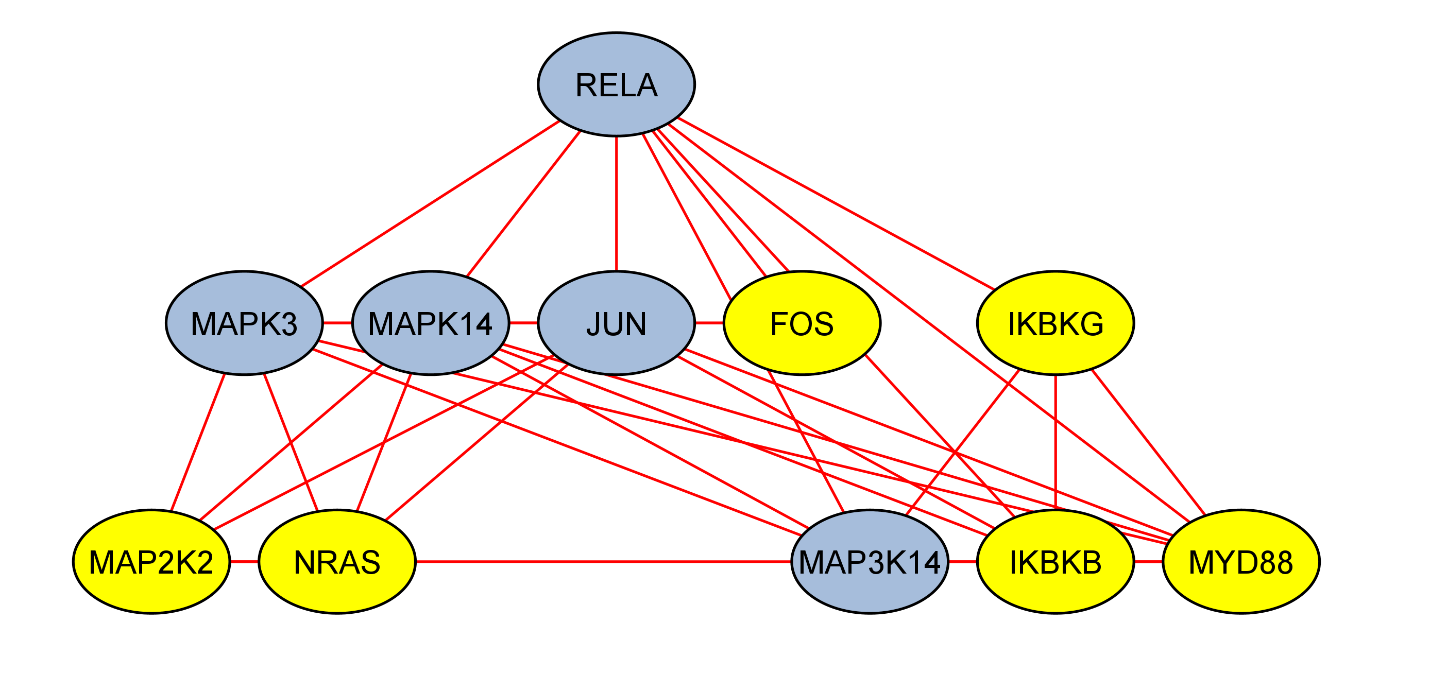

Supplement: Supplementary file 3 [file Image4.TIF]

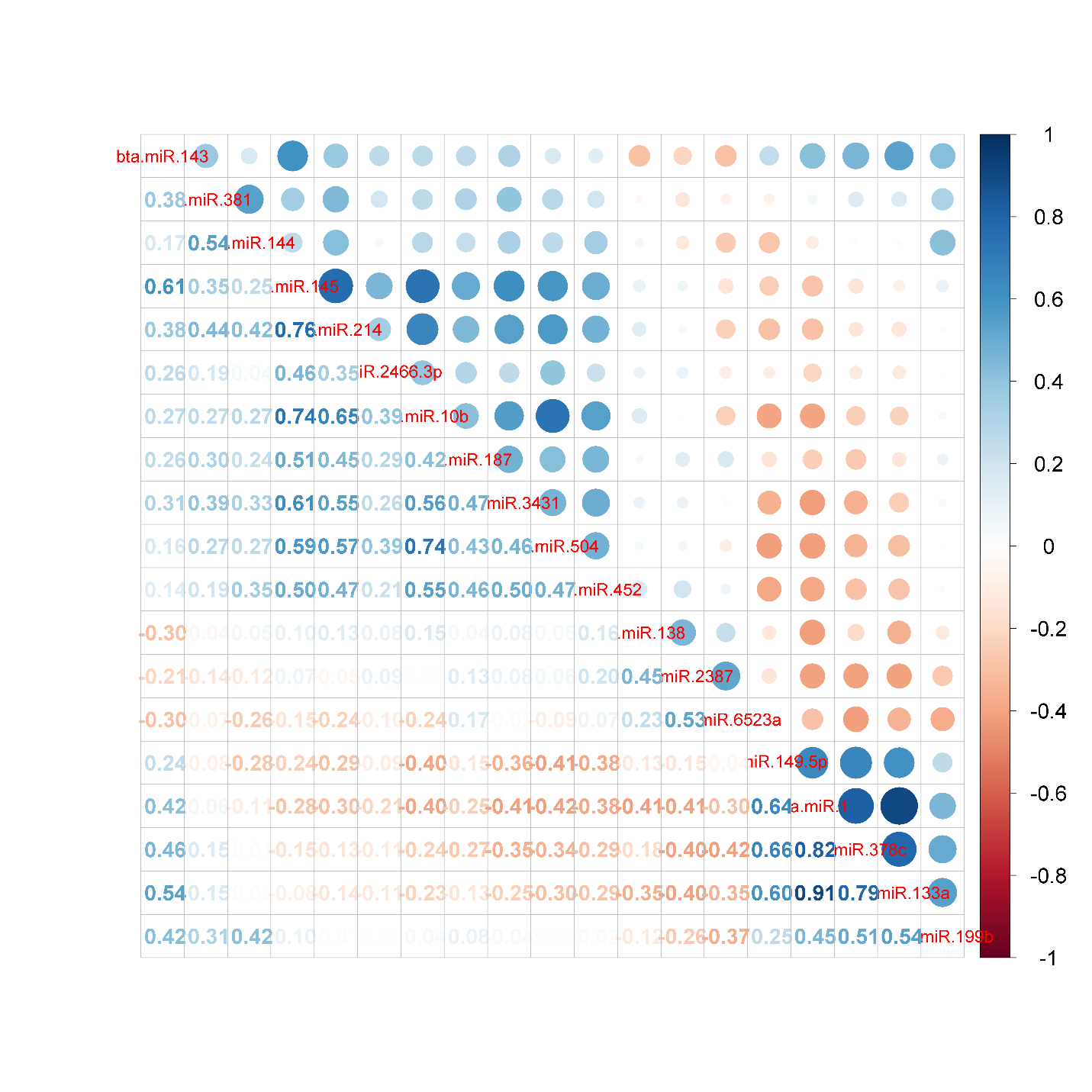

Supplement: Supplementary file 4 [file Image2.TIF]

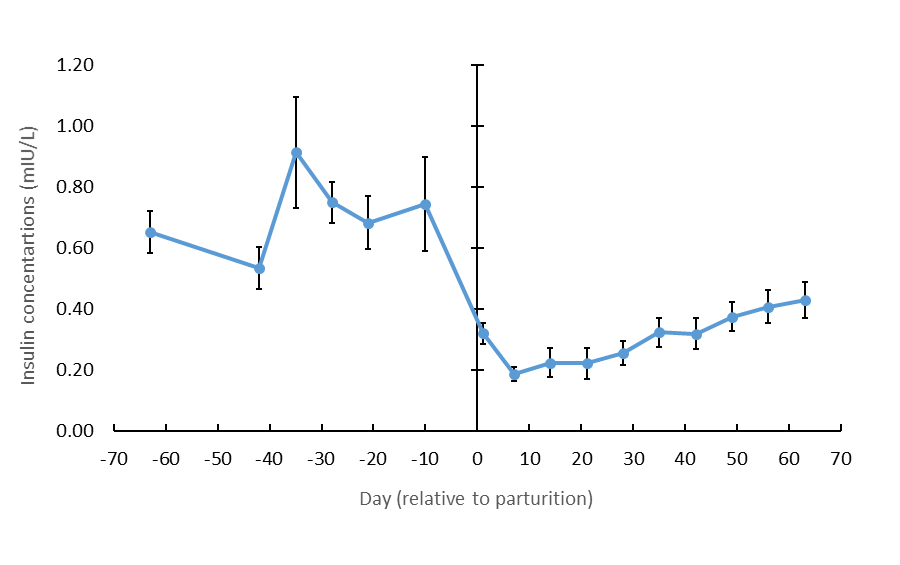

Supplement: Supplementary file 5 [file Image1.TIF]
