# Supplementary material for: Investigating circulating miRNA in transition dairy cows: What miRNAomics tells about metabolic adaptation
Source: Front Genet. 2022 Aug 23;13:946211. doi: 10.3389/fgene.2022.946211 (PMC9445238; doi:10.3389/fgene.2022.946211)
Supplement: Supplementary file 6 [file Table3.DOCX]

Supplementary Table S3. Effect of oil supplementation on plasma miRNA expression profile.

| Time points |  | Treatment^1^ | | | | | | | | | | |
| --- | --- | --- | --- | --- | --- | --- | --- | --- | --- | --- | --- | --- |
|  | CTRL^2^ | |  | EFA^3^ | |  | | CLA^4^ | |  | | EFA+CLA^5^ |
| Day -21 antepartum | - | |  | No Sig^6^ |  | | bta-miR-1 | |  | | No Sig | |
| Day 1 postpartum | - | |  | No Sig |  | | No Sig | |  | | No Sig | |
| Day 28 postpartum | - | |  | No Sig |  | | No Sig | |  | | No Sig | |
| Day 63 postpartum | - | |  | No Sig |  | | No Sig | |  | | No Sig | |

1 Cows were supplemented daily with various fatty acid treatments including coconut oil (CTRL), or mixture of linseed, safflower oil (EFA), Lutalin® (CLA, c9, t11 and t10, c12), and EFA+CLA. At each time point, treatment groups were compared to the CTRL. No significant difference was observed between the treatments, except for bta-miR-1 (log2 (fold change (FC)) = -5.65, false discovery rate (FDR) < 0.001) that was found to be differentially expressed between the CTRL and the CLA group at day -21 AP.

2 Control group (CTRL)

3 Essential fatty acids (EFA)

4 Conjugated linoleic acid (CLA)

5 Mixture of EFA and CLA (EFA+CLA)

6 No significant miRNA found compared to CTRL
